# OptiFit: an Improved Method for Fitting Amplicon Sequences to Existing OTUs

**DOI:** 10.1128/msphere.00916-21

**Published:** 2022-02-02

**Authors:** Kelly L. Sovacool, Sarah L. Westcott, M. Brodie Mumphrey, Gabrielle A. Dotson, Patrick D. Schloss

**Affiliations:** a Department of Computational Medicine and Bioinformatics, University of Michigan, Ann Arbor, Michigan, USA; b Department of Microbiology and Immunology, University of Michigan, Ann Arbor, Michigan, USA; c Center for Computational Medicine and Bioinformatics, University of Michigan, Ann Arbor, Michigan, USA; University of Wisconsin—Madison

**Keywords:** 16S rRNA gene, bioinformatics, clustering, metagenomics, microbial ecology, microbiome

## Abstract

Assigning amplicon sequences to operational taxonomic units (OTUs) is an important step in characterizing microbial communities across large data sets. A notable difference between *de novo* clustering and database-dependent reference clustering methods is that OTU assignments from *de novo* methods may change when new sequences are added. However, one may wish to incorporate new samples to previously clustered data sets without clustering all sequences again, such as when comparing across data sets or deploying machine learning models. Existing reference-based methods produce consistent OTUs but only consider the similarity of each query sequence to a single reference sequence in an OTU, resulting in assignments that are worse than those generated by *de novo* methods. To provide an efficient method to fit sequences to existing OTUs, we developed the OptiFit algorithm. Inspired by the *de novo* OptiClust algorithm, OptiFit considers the similarity of all pairs of reference and query sequences to produce OTUs of the best possible quality. We tested OptiFit using four data sets with two strategies: (i) clustering to a reference database and (ii) splitting the data set into a reference and query set, clustering the references using OptiClust, and then clustering the queries to the references. The result is an improved implementation of reference-based clustering. OptiFit produces OTUs of a quality similar to that of OptiClust at faster speeds when using the split data set strategy. OptiFit provides a suitable option for users requiring consistent OTU assignments at the same quality as afforded by *de novo* clustering methods.

**IMPORTANCE** Advancements in DNA sequencing technology have allowed researchers to affordably generate millions of sequence reads from microorganisms in diverse environments. Efficient and robust software tools are needed to assign microbial sequences into taxonomic groups for characterization and comparison of communities. The OptiClust algorithm produces high-quality groups by comparing sequences to each other, but the assignments can change when new sequences are added to a data set, making it difficult to compare different studies. Other approaches assign sequences to groups by comparing them to sequences in a reference database to produce consistent assignments, but the quality of the groups produced is reduced compared to that with OptiClust. We developed OptiFit, a new reference-based algorithm that produces consistent yet high-quality assignments like OptiClust. OptiFit allows researchers to compare microbial communities across different studies or add new data to existing studies without sacrificing the quality of the group assignments.

## INTRODUCTION

Amplicon sequencing is a mainstay of microbial ecology. Researchers can affordably generate millions of sequences to characterize the composition of hundreds of samples from microbial communities without the need for culturing. In many analysis pipelines, 16S rRNA gene sequences are assigned to operational taxonomic units (OTUs) to facilitate comparison of taxonomic composition between communities to avoid the need for taxonomic classification. A distance threshold of 3% (or sequence similarity of 97%) is commonly used to cluster sequences into OTUs based on pairwise comparisons of the sequences within the data set. The method chosen for clustering affects the quality of OTU assignments and thus may impact downstream analyses of community composition (1–3). OTU quality can be conceptualized as how well the OTU assignments match the definition set by the distance threshold, i.e., whether sequence pairs that are at least as similar as the distance threshold are assigned to the same OTU and sequence pairs that are more dissimilar than the distance threshold are assigned to different OTUs.

There are two main categories of OTU clustering algorithms: *de novo* and reference based. OptiClust is a *de novo* clustering algorithm which uses the distance score between all pairs of sequences in the data set to cluster them into OTUs by maximizing the Matthews correlation coefficient (MCC) (1). This approach takes into account the distances between all pairs of sequences when assigning query sequences to OTUs, in contrast to other *de novo* methods such as the greedy clustering algorithms implemented in USEARCH and VSEARCH (4, 5). In methods employing greedy clustering algorithms, only the distance between each sequence and a representative centroid sequence in the OTU is considered while clustering. As a result, distances between pairs of sequences in the same OTU are frequently larger than the specified threshold, i.e., they are false positives. In contrast, the OptiClust algorithm takes into account the distance between all pairs of sequences when considering how to cluster sequences into OTUs and is thus less willing to take on false positives.

A limitation of *de novo* clustering is that different OTU assignments will be produced when new sequences are added to a data set, making it difficult to use *de novo* clustering to compare OTUs between different studies. Furthermore, since *de novo* clustering requires calculating and comparing distances between all sequences in a data set, the execution time can be slow and memory requirements can be prohibitive for very large data sets. Reference clustering attempts to overcome the limitations of *de novo* clustering methods by using a representative set of sequences from a database, with each reference sequence seeding an OTU. Commonly, the Greengenes set of representative full-length sequences clustered at 97% similarity is used as the reference with VSEARCH (5–7). Query sequences are then clustered into OTUs based on their similarity to the reference sequences. Any query sequences that are not within the distance threshold to any of the reference sequences are either thrown out (closed reference clustering) or clustered *de novo* to create additional OTUs (open reference clustering). While reference-based clustering is generally fast, it is limited by the diversity of the reference database. Novel sequences in the sample will be lost in closed reference mode if they are not represented by a similar sequence in the database. We previously found that the OptiClust *de novo* clustering algorithm created the highest-quality OTU assignments of all clustering methods (1).

To overcome the limitations of current reference-based and *de novo* clustering algorithms while maintaining OTU quality, we developed OptiFit, a reference-based clustering algorithm. While other tools represent reference OTUs with a single sequence, OptiFit uses all sequences in existing OTUs as the reference and fits new sequences to those reference OTUs. In contrast to other tools, OptiFit considers all pairwise distance scores between reference and query sequences when assigning sequences to OTUs in order to produce OTUs of the highest possible quality. In this study, we tested the OptiFit algorithm with the reference as a public database (e.g., Greengenes) or *de novo* OTUs generated using a reference set from the full data set and compared the performance to those of existing tools. To evaluate the OptiFit algorithm and compare it to existing methods, we used four published data sets isolated from soil (8), marine (9), mouse gut (10), and human gut (11) samples. OptiFit is available within the mothur software program.

## RESULTS

### The OptiFit algorithm.

OptiFit leverages the method employed by OptiClust of iteratively assigning sequences to OTUs to produce the highest-quality OTUs possible and extends this method for reference-based clustering. OptiClust first seeds each sequence into its own OTU as a singleton. Then for each sequence, OptiClust considers whether the sequence should move to a different OTU or remain in its current OTU, choosing the option that results in a better MCC score (1). The MCC uses all values from a confusion matrix and ranges from negative one to one, with a score of one occurring when all sequence pairs are true positives and true negatives, a score of negative one occurring when all pairs are false positives and false negatives, and a score of zero when there are equal numbers of true and false assignments (i.e., no better than random guessing). Sequence pairs that are similar to each other (i.e., within the distance threshold) are counted as true positives if they are clustered into the same OTU and false negatives if they are not in the same OTU. Sequence pairs that are not similar to each other are true negatives if they are not clustered into the same OTU and are false positives if they are in the same OTU. Thus, a pair of sequences is considered correctly assigned when their OTU assignment matches the OTU definition set by the distance threshold. OptiClust iterations continue until the MCC stabilizes or until a maximum number of iterations is reached. This process produces *de novo* OTU assignments with the most optimal MCC given the input sequences.

**FIG 1:**
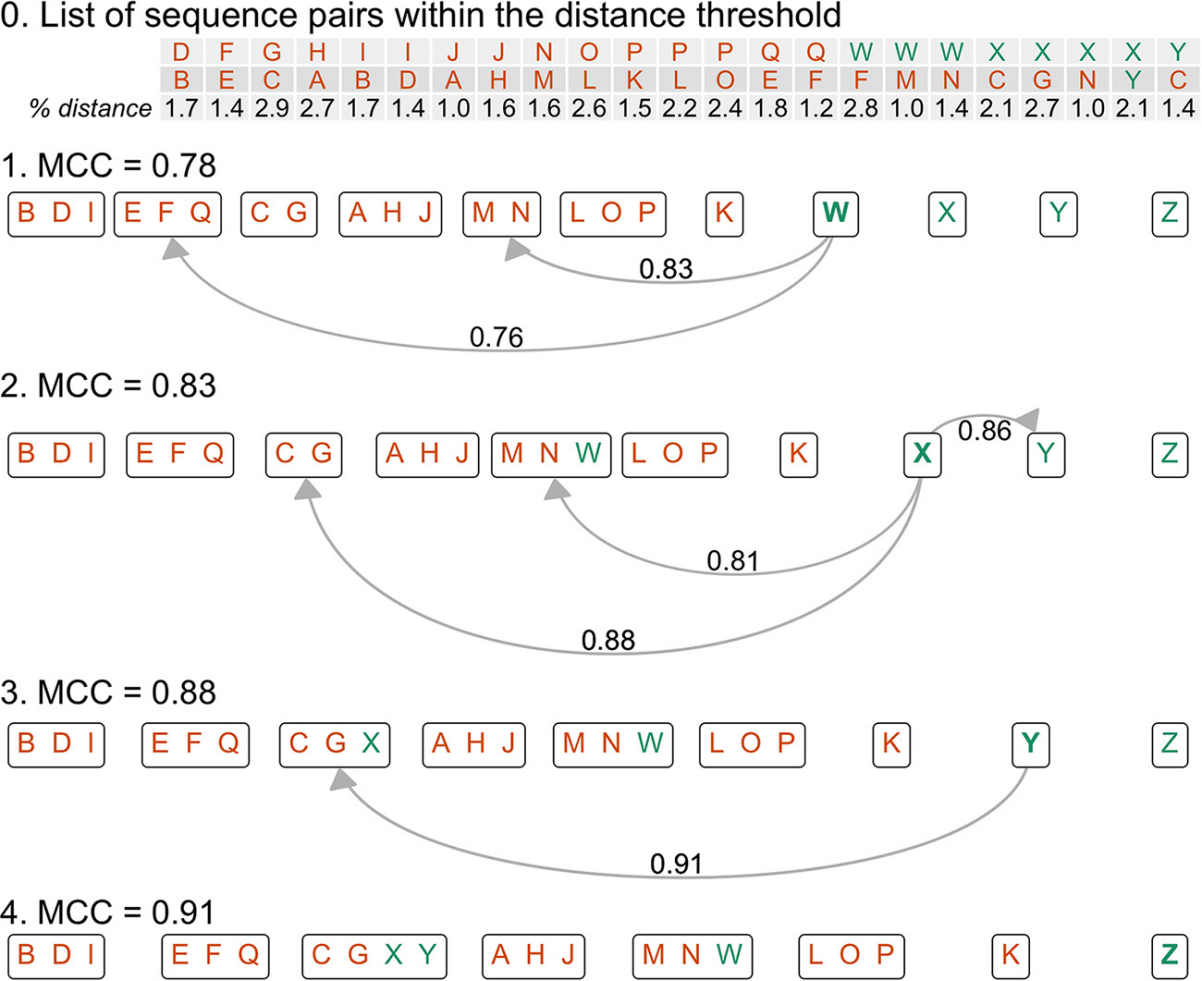
Here, we present a toy example of the OptiFit algorithm fitting query sequences to existing OTUs, given the list of all sequence pairs that are within the distance threshold of 3%. Previously, 50 reference sequences were clustered *de novo* with OptiClust (see the OptiClust supplemental material for reference 1). Reference sequences A through Q (colored orange) were within the distance threshold to at least one other reference sequence; the remaining reference sequences formed additional singleton OTUs (data not shown). The goal of OptiFit is to assign the query sequences W through Z (colored green) to the reference OTUs. Here, there are 50 reference sequences and 4 query sequences which make 1,431 sequence pairs, of which 23 pairs are within the 3% distance threshold. Initially (step 1), OptiFit places each query sequence in its own OTU, resulting in 14 true positives, 9 false negatives, 0 false positives, and 1,408 true negatives for an MCC score of 0.78. Then, for each query sequence (in boldface), OptiFit determines what the new MCC score would be if that sequence were moved to one of the OTUs containing at least one other similar sequence (steps 2 to 4). The sequence is then moved to the OTU which would result in the best MCC score. OptiFit stops iterating over sequences once the MCC score stabilizes. In this example, only one iteration over each sequence was needed. Note that sequence Z was dissimilar to all other sequences and thus remained a singleton. The final MCC score is 0.91, with 20 true positives, 3 false negatives, 1 false positive, and 1,407 true negatives.

OptiFit begins where OptiClust ends, starting with a list of reference OTUs and their sequences, a list of query sequences to cluster to the reference OTUs, and the sequence pairs that are within the distance threshold (e.g., 0.03) (Fig. 1). Initially, all query sequences are placed into separate OTUs. Then, the algorithm iteratively reassigns the query sequences to the reference OTUs to optimize the MCC. Alternatively, a sequence will remain unassigned if the MCC value is maximized when the sequence is a singleton rather than clustered into a reference OTU. All query and reference sequence pairs are considered when calculating the MCC. This process is repeated until the MCC changes by no more than 0.0001 (default) or until a maximum number of iterations is reached (default: 100). In the closed reference mode, any query sequences that cannot be clustered into reference OTUs are discarded, and the results only contain OTUs that exist in the original reference. In the open reference mode, unassigned query sequences are clustered *de novo* using OptiClust to generate new OTUs. The final MCC is reported with the best OTU assignments. There are two strategies for generating OTUs with OptiFit: (i) cluster the query sequences to reference OTUs generated by *de novo* clustering an independent database and (ii) split the data set into a reference and query fraction, cluster the reference sequences *de novo*, and then cluster the query sequences to the reference OTUs.

### Reference clustering with public databases.

**FIG 2:**
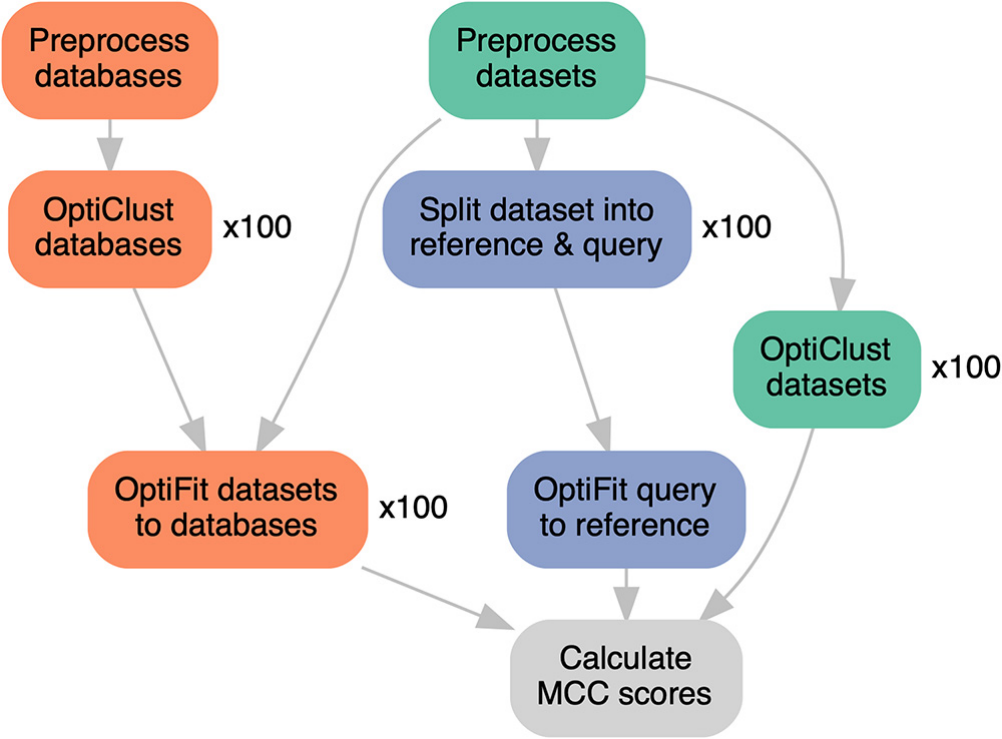
Reference sequences from Greengenes, the RDP, and SILVA were downloaded, preprocessed with mothur by trimming to the V4 region, and clustered *de novo* with OptiClust for 100 repetitions. Data sets from human, marine, mouse, and soil microbiomes were downloaded, preprocessed with mothur by aligning to the SILVA V4 reference alignment, and then clustered *de novo* with OptiClust for 100 repetitions. Individual data sets were fit to reference databases with OptiFit; OptiFit was repeated 100 times for each data set and database combination. Data sets were also randomly split into reference and query fractions, and the query sequences were fit to the reference sequences with OptiFit for 100 repetitions. The final MCC score was reported for all OptiClust and OptiFit repetitions.

**FIG 3:**
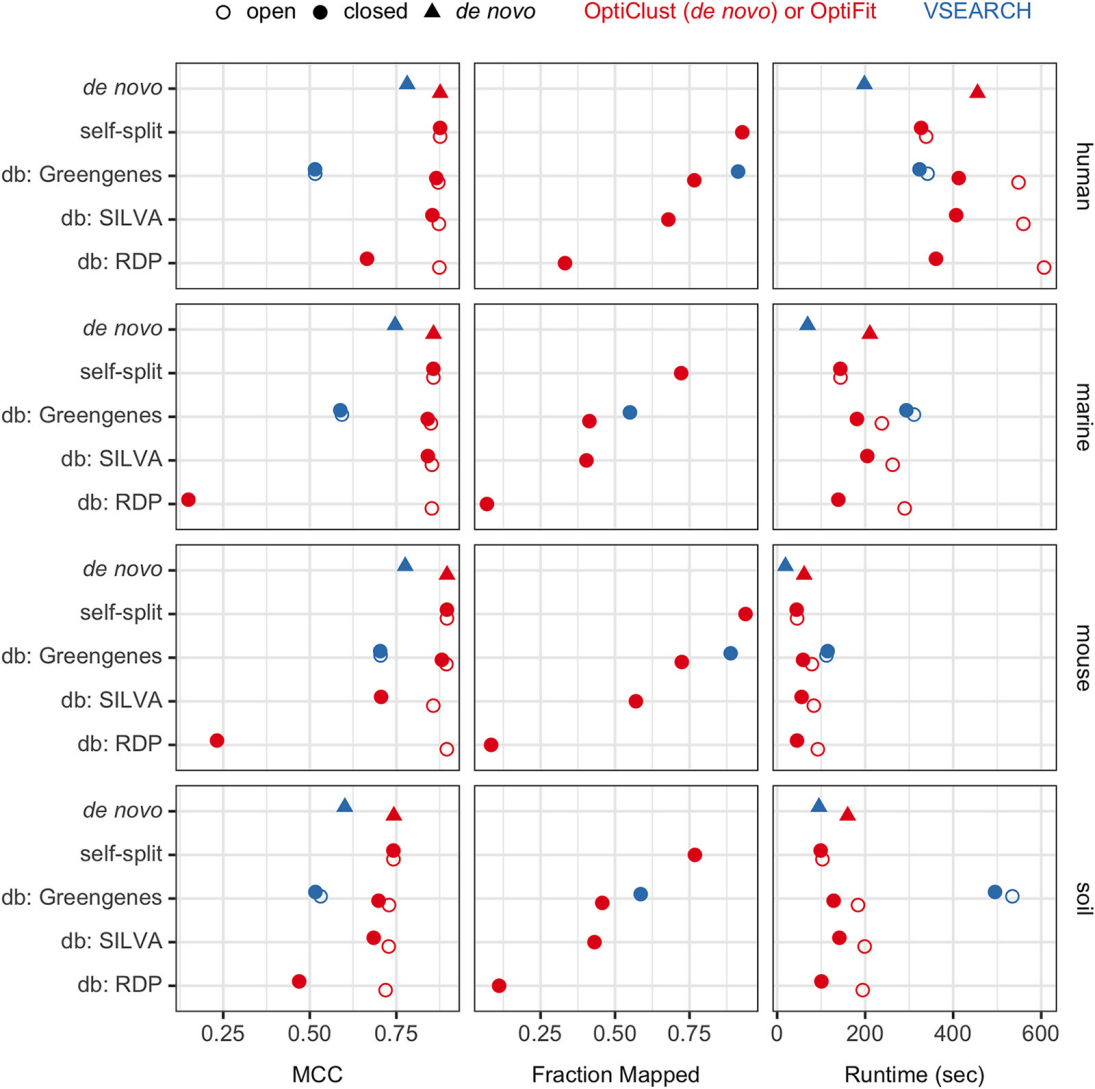
The median MCC score, fraction of query sequences that mapped in closed reference clustering, and runtime in seconds from repeating each clustering method 100 times. Each data set underwent three clustering strategies: (i) *de novo* clustering of the whole data set using OptiClust, (ii) splitting the data set with 50% of the sequences as a reference set and the other 50% as a query set, clustering the references using OptiClust, and then clustering the query sequences to the reference OTUs with OptiFit, and (iii) clustering the data set to a reference database (Greengenes, SILVA, or RDP). Reference-based clustering was repeated with open and closed modes. For additional comparison, VSEARCH was used for *de novo* and reference-based clustering against the Greengenes database.

To test how OptiFit performs for reference-based clustering, we clustered each data set to three databases of reference OTUs: the Greengenes database v13_8_99 (6), the SILVA nonredundant database v132 (12), and the Ribosomal Database Project (RDP) v16 (13). Reference OTUs for each database were created by performing *de novo* clustering with OptiClust at a distance threshold of 3% using the V4 region of each sequence (Fig. 2). After trimming to the V4 region, the databases contained 174,979, 16,192, and 173,648 unique sequences and produced *de novo* MCC scores of 0.72, 0.74, and 0.73 for Greengenes, RDP, and SILVA, respectively. Clustering query sequences with OptiFit to Greengenes and SILVA in closed reference mode performed similarly, with median MCC scores of 0.85 and 0.77, respectively, while the median MCC was 0.35 when clustering to RDP (Fig. 3; “db: Greengenes,” “db: SILVA,” and “db: RDP”). For comparison, clustering data sets with OptiClust produced an average MCC score of 0.86 (Fig. 3; “*de novo*”). This gap in OTU quality mostly disappeared when clustering in open reference mode, which produced median MCCs of 0.86 with Greengenes, 0.86 with SILVA, and 0.86 with the RDP. Thus, open reference OptiFit produced OTUs of a quality very similar to that of *de novo* clustering with OptiClust, and closed reference OptiFit followed closely behind as long as a suitable reference database was chosen.

Since closed reference clustering does not cluster query sequences that could not be clustered into reference OTUs, an additional measure of clustering performance to consider is the fraction of query sequences that were able to be clustered. On average, more sequences were clustered with Greengenes as the reference (59%) than with SILVA (50%) or with the RDP (9.7%) (Fig. 3). This mirrored the result reported above that Greengenes produced better OTUs in terms of MCC score than either SILVA or RDP. Note that *de novo* and open reference clustering methods always cluster 100% of sequences into OTUs. The database chosen affects the final closed reference OTU assignments considerably in terms of both MCC score and fraction of query sequences that could be clustered into the reference OTUs.

Despite the drawbacks, closed reference methods have been used when fast execution speed is required, such as when using very large data sets (14). To compare performance in terms of speed, we repeated each OptiFit and OptiClust run 100 times and measured the execution time. Across all data set and database combinations, closed reference OptiFit outperformed both OptiClust and open reference OptiFit (Fig. 3). For example, with the human data set fit to SILVA reference OTUs, the average run times in seconds were 406.8 for closed reference OptiFit, 455.3 for *de novo* clustering the data set, and 559.4 for open reference OptiFit. Thus, the OptiFit algorithm continues the precedent that closed reference clustering sacrifices OTU quality for execution speed.

To compare to the reference clustering methods used by QIIME2, we clustered each data set with VSEARCH against the Greengenes database of OTUs previously clustered at 97% sequence similarity. Each reference OTU from the Greengenes 97% database contains one reference sequence, and VSEARCH maps sequences to the reference based on each individual query sequence’s similarity to the single reference sequence. In contrast, OptiFit accepts reference OTUs which each may contain multiple sequences, and the sequence similarity between all query and reference sequences is considered when assigning sequences to OTUs. In closed reference mode, OptiFit produced 27.2% higher-quality OTUs than VSEARCH in terms of MCC score, but VSEARCH was able to cluster 24.9% more query sequences than OptiFit to the Greengenes reference database (Fig. 3). This is because VSEARCH only considers the distances between each query sequence to the single reference sequence, while OptiFit considers the distances between all pairs of reference and query sequences in an OTU. When open reference clustering, OptiFit produced higher-quality OTUs than VSEARCH against the Greengenes database, with median MCC scores of 0.86 and 0.56, respectively. In terms of run time, OptiFit outperformed VSEARCH in both closed and open reference mode by 53.6% and 44.0% on average, respectively. Thus, the more stringent OTU definition employed by OptiFit, which prefers the query sequence to be similar to all other sequences in the OTU rather than to only one sequence, resulted in fewer sequences being clustered to reference OTUs than when using VSEARCH but caused OptiFit to outperform VSEARCH in terms of both OTU quality and execution time.

### Reference clustering with split data sets.

When performing reference clustering against public databases, the database chosen greatly affects the quality of OTUs produced. OTU quality may be poor when the reference database consists of sequences that are too unrelated to the samples of interest, such as when samples contain novel populations. While *de novo* clustering overcomes the quality limitations of reference clustering to databases, OTU assignments are not consistent when new sequences are added. Researchers may wish to cluster new sequences to existing OTUs or to compare OTUs across studies. To determine how well OptiFit performs for clustering new sequences to existing OTUs, we employed a split data set strategy, where each data set was randomly split into a reference fraction and a query fraction. Reference sequences were clustered *de novo* with OptiClust, and then query sequences were clustered to the *de novo* OTUs with OptiFit.

First, we tested whether OptiFit performed as well as *de novo* clustering when using the split data set strategy with half of the sequences selected for the reference by a simple random sample (a 50% split) (Fig. 3; “self-split”). OTU quality was similar to that from OptiClust regardless of mode (0.031% difference in median MCC). In closed reference mode, OptiFit was able to cluster 84.9% of query sequences to reference OTUs with the split strategy, a great improvement over the average 59% of sequences clustered to the Greengenes database. In terms of run time, closed and open reference OptiFit performed faster than OptiClust on whole data sets by 39.6% and 36.8%, respectively. Random access memory (RAM) usages were similar, with OptiFit requiring slightly more RAM in gigabytes than OptiClust. Open and closed reference OptiFit required 1.8% and 1.2% more RAM than OptiClust, respectively (data not shown). The split data set strategy also performed 6.7% faster than the database strategy in closed reference mode and 65.5% faster in open reference mode. Thus, reference clustering with the split data set strategy creates OTUs of as high a quality as *de novo* clustering yet at a faster run time, and it fits far more query sequences than the database strategy.

**FIG 4:**
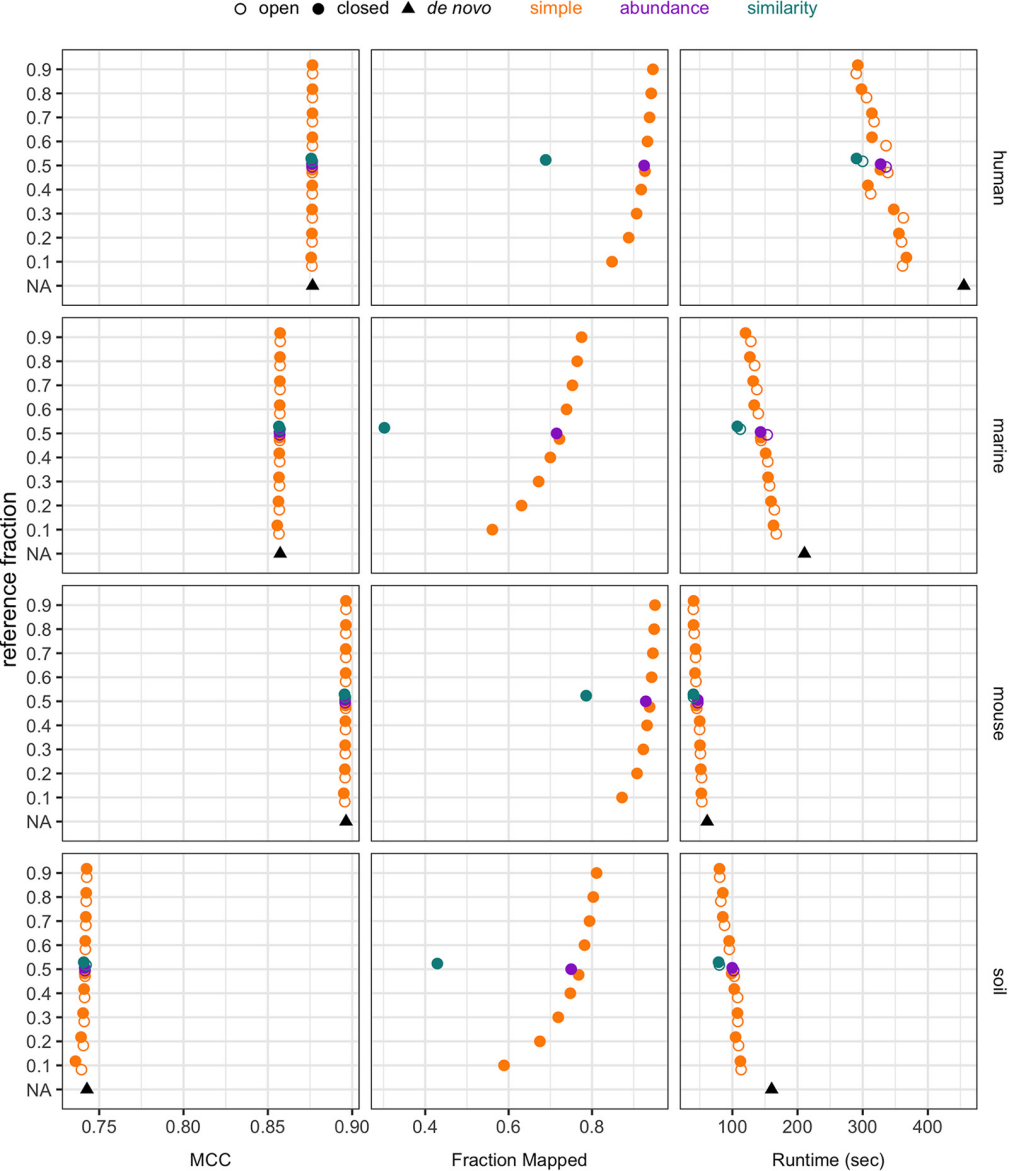
The median MCC score, fraction of query sequences that mapped in closed reference clustering, and runtime in seconds from repeating each clustering method 100 times. Each data set was split into reference and query fractions. Reference sequences were selected via a simple random sample, weighting sequences by relative abundance, or weighting by similarity to other sequences in the data set. With the simple random sample method, data set splitting was repeated with reference fractions ranging from 10% to 90% of the data set and for 100 random seeds. *De novo* clustering each data set with OptiClust is also shown for comparison.

While we initially tested this strategy using a 50% split of the data into reference and query fractions, we next investigated whether there was an optimal reference fraction size. To identify the best reference size, reference sets with 10% to 90% of the sequences were created, with the remaining sequences used for the query (Fig. 4). OTU quality was remarkably consistent across reference fraction sizes. For example, splitting the human data set 100 times yielded a coefficient of variation (i.e., the standard deviation divided by the mean) of 0.0018 for the MCC score across all fractions. Run time generally decreased as the reference fraction increased; for the human data set, the median run time was 364.0 s with 10% of sequences in the reference and 290.8 s with 90% of sequences in the reference. The RAM usage was virtually the same across reference fraction sizes, with a coefficient of variation of 0.00089 for the human data set (data not shown). In closed reference mode, the fraction of sequences that mapped increased as the reference size increased; for the human data set, the median fraction mapped was 0.85 with 10% of sequences in the reference and 0.95 with 90% of sequences in the reference. These trends held for the other data sets as well. Thus, the reference fraction did not affect OTU quality in terms of MCC score or the memory usage, but it did affect the run time and the fraction of sequences that mapped during the closed reference clustering.

After testing the split strategy using a simple random sample to select the reference sequences, we investigated other methods of splitting the data. We tested three methods for selecting the fraction of sequences to be used as the reference at a size of 50%: a simple random sample, weighting sequences by relative abundance, and weighting by similarity to other sequences in the data set (Fig. 4). OTU quality in terms of MCC was similar across all three sampling methods (median MCC of 0.86). In closed reference clustering mode, the fractions of sequences that mapped were similar for simple and abundance-weighted sampling (median fractions mapped of 0.85 and 0.84, respectively) but worse for similarity-weighted sampling (median fraction mapped of 0.56). While simple and abundance-weighted sampling produced better-quality OTUs than similarity-weighted sampling, OptiFit performed faster on similarity-weighted samples, with a median runtime of 103.9 s, compared to 135.4 and 134.8 s for simple and abundance-weighted sampling, respectively. Thus, employing more complicated sampling strategies such as abundance-weighted and similarity-weighted sampling did not confer any advantages over selecting the reference via a simple random sample and in fact decreased OTU quality in the case of similarity-weighted sampling.

## DISCUSSION

We developed a new algorithm for clustering sequences to existing OTUs and have demonstrated its suitability for reference-based clustering. OptiFit makes the iterative method employed by OptiClust available for tasks where reference-based clustering is required. We have shown that degrees of OTU quality are similar between OptiClust and OptiFit in open reference mode, regardless of strategy employed. Open reference OptiFit performs slower than OptiClust due to the additional *de novo* clustering step, so users may prefer OptiClust for tasks that do not require reference OTUs.

When clustering to public databases, OTU quality dropped in closed reference mode to different degrees depending on the database and data set source, and no more than half of query sequences were able to be clustered into OTUs across any data set/database combination. This may reflect limitations of reference databases, which are unlikely to contain sequences from novel microbes. This drop in quality was most notable with the RDP reference, which contained only 16,192 sequences, compared to 173,648 sequences in SILVA and 174,979 in Greengenes. Note that Greengenes has not been updated since 2013 at the time of this writing, while SILVA and the RDP are updated regularly. We recommend that users who require an independent reference database opt for large databases with regular updates and good coverage of microbial diversity for their environment. Since OptiClust still performs faster than open reference OptiFit and creates higher-quality OTUs than closed reference OptiFit with the database strategy, we recommend using OptiClust rather than clustering to a database whenever consistent OTUs are not required.

The OptiClust and OptiFit algorithms produced higher-quality OTUs than VSEARCH in open reference, closed reference, and *de novo* modes. However, VSEARCH was able to cluster more sequences to OTUs than OptiFit in closed reference mode. While both OptiFit and VSEARCH use a distance or similarity threshold for determining how to cluster sequences into OTUs, VSEARCH is more permissive than OptiFit regardless of mode. The OptiFit and OptiClust algorithms use all of the sequences to define an OTU, preferring that all pairs of sequences (including reference and query sequences) in an OTU are within the distance threshold in order to maximize the MCC. In contrast, VSEARCH only requires each query sequence to be similar to the single centroid sequence that seeded the OTU, thus allowing pairs of query sequences to be less similar to each other than the threshold specified. Because of this, VSEARCH sacrifices OTU quality by allowing more dissimilar sequences to be clustered into the same OTUs.

When clustering with the split data set strategy, OTU quality was remarkably similar when reference sequences were selected by a simple random sample or weighted by abundance, but quality was slightly worse when sequences were weighted by similarity. We recommend using a simple random sample since the more sophisticated reference selection methods do not offer any benefit. The similarity in OTU quality between OptiClust and OptiFit with this strategy demonstrates the suitability of using OptiFit to cluster sequences to existing OTUs, such as when comparing OTUs across studies. However, when consistent OTUs are not required, we recommend using OptiClust for *de novo* clustering over the split strategy with OptiFit since OptiClust is simpler to execute but performs similarly in terms of both run time and OTU quality.

Unlike existing reference-based methods that cluster query sequences to a single centroid sequence in each reference OTU, OptiFit considers all sequences in each reference OTU when clustering query sequences, resulting in OTUs of a similar high quality as those produced by the *de novo* OptiClust algorithm. Potential applications include clustering sequences to reference databases, comparing taxonomic composition of microbiomes across different studies, and using OTU-based machine learning models to make predictions on new data. OptiFit fills the missing option for clustering query sequences to existing OTUs that does not sacrifice OTU quality for consistency of OTU assignments.

## MATERIALS AND METHODS

### Data processing steps.

We downloaded 16S rRNA gene amplicon sequences from four published data sets isolated from soil (8), marine (9), mouse gut (10), and human gut (11) samples. These data sets contain sequences from the V4 region of the 16S rRNA gene and represent a selection of the broad types of natural communities that microbial ecologists study. We processed the raw sequences using mothur according to the Schloss Lab MiSeq SOP (15) and accompanying study by Kozich et al. (16). These steps included trimming and filtering for quality, aligning to the SILVA reference alignment (12), discarding sequences that aligned outside the V4 region, removing chimeric reads with UCHIME (17), and calculating distances between all pairs of sequences within each data set prior to clustering.

### Reference database clustering.

To generate reference OTUs from public databases, we downloaded sequences from the Greengenes database (v13_8_99) (6), SILVA nonredundant database (v132) (12), and the Ribosomal Database Project (v16) (13). These sequences were processed using the same steps as outlined above followed by clustering sequences into *de novo* OTUs with OptiClust. Processed reads from each of the four data sets were clustered with OptiFit to the reference OTUs generated from each of the three databases. When reference clustering with VSEARCH, processed data sets were clustered directly to the unprocessed Greengenes 97% OTU reference alignment, since this method is how VSEARCH is typically used by the QIIME2 software for reference-based clustering (7, 18).

### Split data set clustering.

For each data set, half of the sequences were selected to be clustered *de novo* into reference OTUs with OptiClust. We used three methods for selecting the subset of sequences to be used as the reference: a simple random sample, weighting sequences by relative abundance, and weighting by similarity to other sequences in the data set. Data set splitting was repeated with 100 random seeds. With the simple random sampling method, data set splitting was also repeated with reference fractions ranging from 10% to 90% of the data set. For each data set split, the remaining query sequences were clustered into the reference OTUs with OptiFit.

### Benchmarking.

OptiClust and OptiFit randomize the order of query sequences prior to clustering and employ a random number generator to break ties when OTU assignments are of equal quality. As a result, they produce slightly different OTU assignments when repeated with different random seeds. To capture any variation in OTU quality or execution time, clustering was repeated with 100 random seeds for each combination of parameters and input data sets. We used the benchmark feature provided by Snakemake to measure the run time of every clustering job. We calculated the MCC on each set of OTUs to quantify the quality of clustering, as described by Westcott and Schloss (1).

### Data availability.

We implemented the analysis workflow in Snakemake (19) and wrote scripts in R (20), Python (21), and GNU bash (22). Software used includes mothur v1.47.0 (23), VSEARCH v2.15.2 (5), the Tidyverse metapackage (24), R Markdown (25), ggraph (26), ggtext (27), numpy (28), the SRA toolkit (29), and conda (30). The complete workflow and supporting files required to reproduce this study are available at https://github.com/SchlossLab/Sovacool_OptiFit_mSphere_2022.

## References

[1] Westcott SL, Schloss PD. 2017. OptiClust, an improved method for assigning amplicon-based sequence data to operational taxonomic units. mSphere 2:e00073-17. doi:10.1128/mSphereDirect.00073-17. 28289728 PMC5343174

[2] Schloss PD. 2016. Application of a database-independent approach to assess the quality of operational taxonomic unit picking methods. mSystems 1:e00027-16. doi:10.1128/mSystems.00027-16. PMC5069744 27832214

[3] Westcott SL, Schloss PD. 2015. De novo clustering methods outperform reference-based methods for assigning 16S rRNA gene sequences to operational taxonomic units. PeerJ 3:e1487. doi:10.7717/peerj.1487. 26664811 PMC4675110

[4] Edgar RC. 2010. Search and clustering orders of magnitude faster than BLAST. Bioinformatics 26:2460–2461. doi:10.1093/bioinformatics/btq461. 20709691

[5] Rognes T, Flouri T, Nichols B, Quince C, Mahé F. 2016. VSEARCH: a versatile open source tool for metagenomics. PeerJ 4:e2584. doi:10.7717/peerj.2584. 27781170 PMC5075697

[6] DeSantis TZ, Hugenholtz P, Larsen N, Rojas M, Brodie EL, Keller K, Huber T, Dalevi D, Hu P, Andersen GL. 2006. Greengenes, a chimera-checked 16S rRNA gene database and workbench compatible with ARB. Appl Environ Microbiol 72:5069–5072. doi:10.1128/AEM.03006-05. 16820507 PMC1489311

[7] QIIME 2 development team. 2021. Clustering sequences into OTUs using Q2-vsearch QIIME 2 2021.2.0 documentation. https://docs.qiime2.org/2021.2/tutorials/otu-clustering/.

[8] Johnston ER, Rodriguez-R LM, Luo C, Yuan MM, Wu L, He Z, Schuur EAG, Luo Y, Tiedje JM, Zhou J, Konstantinidis KT. 2016. Metagenomics reveals pervasive bacterial populations and reduced community diversity across the Alaska tundra ecosystem. Front Microbiol 7:579. doi:10.3389/fmicb.2016.00579. 27199914 PMC4842900

[9] Henson MW, Pitre DM, Weckhorst JL, Lanclos VC, Webber AT, Thrash JC. 2016. Artificial seawater media facilitate cultivating members of the microbial majority from the Gulf of Mexico. mSphere 1:e00124-16. doi:10.1128/mSphere.00124-16. 27303734 PMC4894692

[10] Schloss PD, Schubert AM, Zackular JP, Iverson KD, Young VB, Petrosino JF. 2012. Stabilization of the murine gut microbiome following weaning. Gut Microbes 3:383–393. doi:10.4161/gmic.21008. 22688727 PMC3463496

[11] Baxter NT, Ruffin MT, Rogers MAM, Schloss PD. 2016. Microbiota-based model improves the sensitivity of fecal immunochemical test for detecting colonic lesions. Genome Med 8:37. doi:10.1186/s13073-016-0290-3. 27056827 PMC4823848

[12] Quast C, Pruesse E, Yilmaz P, Gerken J, Schweer T, Yarza P, Peplies J, Glöckner FO. 2013. The SILVA ribosomal RNA gene database project: improved data processing and web-based tools. Nucleic Acids Res 41:D590–D596. doi:10.1093/nar/gks1219. 23193283 PMC3531112

[13] Cole JR, Wang Q, Fish JA, Chai B, McGarrell DM, Sun Y, Brown CT, Porras-Alfaro A, Kuske CR, Tiedje JM. 2014. Ribosomal Database Project: data and tools for high throughput rRNA analysis. Nucleic Acids Res 42:D633–D642. doi:10.1093/nar/gkt1244. 24288368 PMC3965039

[14] Navas-Molina JA, Peralta-Sánchez JM, González A, McMurdie PJ, Vázquez-Baeza Y, Xu Z, Ursell LK, Lauber C, Zhou H, Song SJ, Huntley J, Ackermann GL, Berg-Lyons D, Holmes S, Caporaso JG, Knight R. 2013. Advancing our understanding of the human microbiome using QIIME. Methods Enzymol 531:371–444. doi:10.1016/B978-0-12-407863-5.00019-8. 24060131 PMC4517945

[15] Schloss PD, Westcott SL. MiSeq SOP. https://mothur.org/wiki/miseq_sop/.

[16] Kozich JJ, Westcott SL, Baxter NT, Highlander SK, Schloss PD. 2013. Development of a dual-index sequencing strategy and curation pipeline for analyzing amplicon sequence data on the MiSeq Illumina sequencing platform. Appl Environ Microbiol 79:5112–5120. doi:10.1128/AEM.01043-13. 23793624 PMC3753973

[17] Edgar RC, Haas BJ, Clemente JC, Quince C, Knight R. 2011. UCHIME improves sensitivity and speed of chimera detection. Bioinformatics 27:2194–2200. doi:10.1093/bioinformatics/btr381. 21700674 PMC3150044

[18] Bolyen E, Rideout JR, Dillon MR, Bokulich NA, Abnet CC, Al-Ghalith GA, Alexander H, Alm EJ, Arumugam M, Asnicar F, Bai Y, Bisanz JE, Bittinger K, Brejnrod A, Brislawn CJ, Brown CT, Callahan BJ, Caraballo-Rodríguez AM, Chase J, Cope EK, Da Silva R, Diener C, Dorrestein PC, Douglas GM, Durall DM, Duvallet C, Edwardson CF, Ernst M, Estaki M, Fouquier J, Gauglitz JM, Gibbons SM, Gibson DL, Gonzalez A, Gorlick K, Guo J, Hillmann B, Holmes S, Holste H, Huttenhower C, Huttley GA, Janssen S, Jarmusch AK, Jiang L, Kaehler BD, Kang KB, Keefe CR, Keim P, Kelley ST, Knights D, et al. 2019. Reproducible, interactive, scalable and extensible microbiome data science using QIIME 2. Nat Biotechnol 37:852–857. doi:10.1038/s41587-019-0209-9. 31341288 PMC7015180

[19] Köster J, Rahmann S. 2012. Snakemake a scalable bioinformatics workflow engine. Bioinformatics 28:2520–2522. doi:10.1093/bioinformatics/bts480. 22908215

[20] R Core Team. 2020. R: a language and environment for statistical computing. R Foundation for Statistical Computing, Vienna, Austria.

[21] Van Rossum G, Drake FL. 2009. Python 3 reference manual. CreateSpace, Scotts Valley, CA.

[22] GNU Project. Bash reference manual. https://www.gnu.org/software/bash/manual/bash.html.

[23] Schloss PD, Westcott SL, Ryabin T, Hall JR, Hartmann M, Hollister EB, Lesniewski RA, Oakley BB, Parks DH, Robinson CJ, Sahl JW, Stres B, Thallinger GG, Van Horn DJ, Weber CF. 2009. Introducing mothur: open-source, platform-independent, community-supported software for describing and comparing microbial communities. Appl Environ Microbiol 75:7537–7541. doi:10.1128/AEM.01541-09. 19801464 PMC2786419

[24] Wickham H, Averick M, Bryan J, Chang W, McGowan LD, François R, Grolemund G, Hayes A, Henry L, Hester J, Kuhn M, Pedersen TL, Miller E, Bache SM, Müller K, Ooms J, Robinson D, Seidel DP, Spinu V, Takahashi K, Vaughan D, Wilke C, Woo K, Yutani H. 2019. Welcome to the Tidyverse. J Open Source Softw 4:1686. doi:10.21105/joss.01686.

[25] Xie Y, Allaire JJ, Grolemund G. 2018. R Markdown: the definitive guide. Taylor & Francis, CRC Press, Abingdon-on-Thames, United Kingdom.

[26] Pedersen TL. 2021. ggraph: an implementation of grammar of graphics for graphs and networks.

[27] Wilke CO. 2020. ggtext: improved text rendering support for ’Ggplot2.’

[28] Harris CR, Millman KJ, van der Walt SJ, Gommers R, Virtanen P, Cournapeau D, Wieser E, Taylor J, Berg S, Smith NJ, Kern R, Picus M, Hoyer S, van Kerkwijk MH, Brett M, Haldane A, del Río JF, Wiebe M, Peterson P, Gérard-Marchant P, Sheppard K, Reddy T, Weckesser W, Abbasi H, Gohlke C, Oliphant TE. 2020. Array programming with NumPy. Nature 585:357–362. doi:10.1038/s41586-020-2649-2. 32939066 PMC7759461

[29] NCBI. SRA-Tools. https://github.com/ncbi/sra-tools.

[30] Anaconda Inc. 2016. Anaconda documentation.

